# Supplementary material for: A sharp Pif1-dependent threshold separates DNA double-strand breaks from critically short telomeres
Source: eLife. 2017 Aug 3;6:e23783. doi: 10.7554/eLife.23783 (PMC5595431; doi:10.7554/eLife.23783)
Supplement: Supplementary file 1. [file elife-23783-supp1.docx]

**Supplementary File 1A: Sequences of DNA ends**

| **TG repeat** | **Sequence (5’ to 3’)** |
| --- | --- |
| TG_6_ | ACCACA |
| TG_12_ | ACACACCCACAC |
| TG_18_ | ACACCACACCCACACACA |
| TG_22_ | ACACCACACCCACACACACACC |
| TG_26_ | ACACCACACCCACACACACACCCACA |
| TG_30_ | ACACCACACCCACACACACACCCACACCCA |
| TG_34_ | ACACACACACCACACCCACACCCACACACCACAC |
| TG_34v2_ | ACACCACACCCACACACACACCCACACCCACACA |
| TG_38_ | ACACCACACCCACACACACACCCACACCCACACACCAC |
| TG_42_ | ACACACACACCACACCCACACCCACACACCACACCACACACA |
| TG_45_ | ACCCACACACCCACACCCACACACCACACCCACACACACCACACC |
| TG_50_ | ACACACACACCACACCCACACCCACACACCACACCACACACACCACACCC |
| TG_56_ | ACACCCACCACACCCACACACCCACACCCACACACCACACCCACACACACCACACC |
| TG_58_ | ACACACACACCACACCCACACCCACACACCACACCACACACACCACACCCACCACACC |
| TG_66_ | ACACACACACCACACCCACACCCACACACCACACCACACACACCACACCCACCACACCCACACACC |
| TG_67_ | ACACACACACCACACCCACCACACCCACACACCCACACCCACACACCACACCCACACACACCACACC |
| TG_74_ | ACACACACACCACACCCACACCCACACACCACACCACACACACCACACCCACCACACCCACACACCCACACCCA |
| TG_82_ | ACACACACACCACACCCACACCCACACACCACACCACACACACCACACCCACCACACCCACACACCCACACCCACACACCAC |
| TG_26-A_ | ACCACACACCCACACACCACACCCAC |
| TG_36-A_ | ACCACACACCCACACACCACACCCACACACACCACA |
| TG_26-B_ | ACCACACACACCACACCCACACCACA |
| TG_36-B_ | ACCACACACACCACACCCACACCACACCCACACACC |
| TG_26-C_ | ACCACACCACACCCACACACCACACC |
| TG_36-C_ | ACCACACCACACCCACACACCACACCCACACACACC |
| TG_26-(TGTGG)_ | ACCACACCACACCACACCACACCACA |
| TG_36-(TGTGG)_ | ACCACACCACACCACACCACACCACACCACACCACA |
| TG_26-(TG)_ | ACACACACACACACACACACACACAC |
| TG_36-(TG)_ | ACACACACACACACACACACACACACACACACACAC |

**Supplementary File 1B: Yeast strains**

| **Strain** | **Strain** |  |  |
| --- | --- | --- | --- |
| **Number** | **Background** | **Genotype** | **Source** |
| DDY2458 | S288C | *MAT***a***-inc ura3-52 lys2-801 ade2-101 ochre trp1-Δ63 his3-Δ200 leu2-Δ1::GAL1:HO-LEU2 rad52::HIS* *pRAD52-TRP VII-L-ADE2-TG82-HOcs-LYS2* | (Zhang & Durocher, 2010) |
| DDY2476 | S288C | *MAT***a***-inc ura3-52 lys2-801 ade2-101 ochre trp1-Δ63 his3-Δ200 leu2-Δ1::GAL1:HO-LEU2 rad52::HIS* *pRAD52-TRP VII-L-ADE2-TG82-HOcs-LYS2 pif1-m2* | (Zhang & Durocher, 2010) |
| DDY3254 | S288C | *MAT***a***-inc ura3-52 lys2-801 ade2-101 ochre trp1-Δ63 his3-Δ200 leu2-Δ1::GAL1:HO-LEU2 rad52::HIS* *VII-L::URA3-TG18-HOcs-LYS2* | This study |
| DDY3203 | S288C | *MAT***a***-inc ura3-52 lys2-801 ade2-101 ochre trp1-Δ63 his3-Δ200 leu2-Δ1::GAL1:HO-LEU2 rad52::HIS* *VII-L::URA3-TG18-HOcs-LYS2 pif1-m2* | This study |
| DDY3376 | S288C | *MAT***a***-inc ura3-52 lys2-801 ade2-101 ochre trp1-Δ63 his3-Δ200 leu2-Δ1::GAL1:HO-LEU2 rad52::HIS* *VII-L::URA3-TG34-HOcs-LYS2* | This study |
| DDY2986 | S288C | *MAT***a***-inc ura3-52 lys2-801 ade2-101 ochre trp1-Δ63 his3-Δ200 leu2-Δ1::GAL1:HO-LEU2 rad52::HIS* *VII-L::URA3-TG34-HOcs-LYS2 pif1-m2* | This study |
| DDY2985 | S288C | *MAT***a***-inc ura3-52 lys2-801 ade2-101 ochre trp1-Δ63 his3-Δ200 leu2-Δ1::GAL1:HO-LEU2 rad52::HIS* *VII-L::URA3-TG45-HOcs-LYS2* | This study |
| DDY2987 | S288C | *MAT***a***-inc ura3-52 lys2-801 ade2-101 ochre trp1-Δ63 his3-Δ200 leu2-Δ1::GAL1:HO-LEU2 rad52::HIS* *VII-L::URA3-TG45-HOcs-LYS2 pif1-m2* | This study |
| DDY3129 | S288C | *MAT***a***-inc ura3-52 lys2-801 ade2-101 ochre trp1-Δ63 his3-Δ200 leu2-Δ1::GAL1:HO-LEU2 rad52::HIS* *VII-L::URA3-TG56-HOcs-LYS2* | This study |
| DDY3130 | S288C | *MAT***a***-inc ura3-52 lys2-801 ade2-101 ochre trp1-Δ63 his3-Δ200 leu2-Δ1::GAL1:HO-LEU2 rad52::HIS* *VII-L::URA3-TG56-HOcs-LYS2 pif1-m2* | This study |
| DDY2988 | S288C | *MAT***a***-inc ura3-52 lys2-801 ade2-101 ochre trp1-Δ63 his3-Δ200 leu2-Δ1::GAL1:HO-LEU2 rad52::HIS* *VII-L::URA3-TG67-HOcs-LYS2* | This study |
| DDY2990 | S288C | *MAT***a***-inc ura3-52 lys2-801 ade2-101 ochre trp1-Δ63 his3-Δ200 leu2-Δ1::GAL1:HO-LEU2 rad52::HIS* *VII-L::URA3-TG67-HOcs-LYS2 pif1-m2* | This study |
| DDY2472 | S288C | *MAT***a***-inc ura3-52 lys2-801 ade2-101 ochre trp1-Δ63 his3-Δ200 leu2-Δ1::GAL1:HO-LEU2 rad52::HIS* *VII-L-ADE2-TG82-HOcs-LYS2* | This study |
| DDY2556 | S288C | *MAT***a***-inc ura3-52 lys2-801 ade2-101 ochre trp1-Δ63 his3-Δ200 leu2-Δ1::GAL1:HO-LEU2 rad52::HIS* *VII-L-ADE2-TG82-HOcs-LYS2 -m2* | This study |
| DDY3204 | S288C | *MAT***a***-inc ura3-52 lys2-801 ade2-101 ochre trp1-Δ63 his3-Δ200 leu2-Δ1::GAL1:HO-LEU2 rad52::HIS* *VII-L::URA3-TG22-HOcs-LYS2* | This study |
| DDY3205 | S288C | *MAT***a***-inc ura3-52 lys2-801 ade2-101 ochre trp1-Δ63 his3-Δ200 leu2-Δ1::GAL1:HO-LEU2 rad52::HIS* *VII-L::URA3-TG22-HOcs-LYS2 pif1-m2* | This study |
| DDY3206 | S288C | *MAT***a***-inc ura3-52 lys2-801 ade2-101 ochre trp1-Δ63 his3-Δ200 leu2-Δ1::GAL1:HO-LEU2 rad52::HIS* *VII-L::URA3-TG26-HOcs-LYS2* | This study |
| DDY3207 | S288C | *MAT***a***-inc ura3-52 lys2-801 ade2-101 ochre trp1-Δ63 his3-Δ200 leu2-Δ1::GAL1:HO-LEU2 rad52::HIS* *VII-L::URA3-TG26-HOcs-LYS2 pif1-m2* | This study |
| DDY3208 | S288C | *MAT***a***-inc ura3-52 lys2-801 ade2-101 ochre trp1-Δ63 his3-Δ200 leu2-Δ1::GAL1:HO-LEU2 rad52::HIS* *VII-L::URA3-TG30-HOcs-LYS2* | This study |
| DDY3209 | S288C | *MAT***a***-inc ura3-52 lys2-801 ade2-101 ochre trp1-Δ63 his3-Δ200 leu2-Δ1::GAL1:HO-LEU2 rad52::HIS* *VII-L::URA3-TG30-HOcs-LYS2 pif1-m2* | This study |
| DDY3210 | S288C | *MAT***a***-inc ura3-52 lys2-801 ade2-101 ochre trp1-Δ63 his3-Δ200 leu2-Δ1::GAL1:HO-LEU2 rad52::HIS* *VII-L::URA3-TG34v2-HOcs-LYS2* | This study |
| DDY3211 | S288C | *MAT***a***-inc ura3-52 lys2-801 ade2-101 ochre trp1-Δ63 his3-Δ200 leu2-Δ1::GAL1:HO-LEU2 rad52::HIS* *VII-L::URA3-TG34v2-HOcs-LYS2 pif1-m2* | This study |
| DDY3404 | S288C | *MAT***a***-inc ura3-52 lys2-801 ade2-101 ochre trp1-Δ63 his3-Δ200 leu2-Δ1::GAL1:HO-LEU2 rad52::HIS* *VII-L::URA3-TG38-HOcs-LYS2* | This study |
| DDY3406 | S288C | *MAT***a***-inc ura3-52 lys2-801 ade2-101 ochre trp1-Δ63 his3-Δ200 leu2-Δ1::GAL1:HO-LEU2 rad52::HIS* *VII-L::URA3-TG38-HOcs-LYS2 pif1-m2* | This study |
| DDY3275 | S288C | *MAT***a***-inc ura3-52 lys2-801 ade2-101 ochre trp1-Δ63 his3-Δ200 leu2-Δ1::GAL1:HO-LEU2 rad52::HIS* *VII-L::URA3-TG26a-HOcs-LYS2* | This study |
| DDY3276 | S288C | *MAT***a***-inc ura3-52 lys2-801 ade2-101 ochre trp1-Δ63 his3-Δ200 leu2-Δ1::GAL1:HO-LEU2 rad52::HIS* *VII-L::URA3-TG26a-HOcs-LYS2 pif1-m2* | This study |
| DDY3277 | S288C | *MAT***a***-inc ura3-52 lys2-801 ade2-101 ochre trp1-Δ63 his3-Δ200 leu2-Δ1::GAL1:HO-LEU2 rad52::HIS* *VII-L::URA3-TG36a-HOcs-LYS2* | This study |
| DDY3278 | S288C | *MAT***a***-inc ura3-52 lys2-801 ade2-101 ochre trp1-Δ63 his3-Δ200 leu2-Δ1::GAL1:HO-LEU2 rad52::HIS* *VII-L::URA3-TG36a-HOcs-LYS2 pif1-m2* | This study |
| DDY3279 | S288C | *MAT***a***-inc ura3-52 lys2-801 ade2-101 ochre trp1-Δ63 his3-Δ200 leu2-Δ1::GAL1:HO-LEU2 rad52::HIS* *VII-L::URA3-TG26b-HOcs-LYS2* | This study |
| DDY3280 | S288C | *MAT***a***-inc ura3-52 lys2-801 ade2-101 ochre trp1-Δ63 his3-Δ200 leu2-Δ1::GAL1:HO-LEU2 rad52::HIS* *VII-L::URA3-TG26b-HOcs-LYS2 pif1-m2* | This study |
| DDY3281 | S288C | *MAT***a***-inc ura3-52 lys2-801 ade2-101 ochre trp1-Δ63 his3-Δ200 leu2-Δ1::GAL1:HO-LEU2 rad52::HIS* *VII-L::URA3-TG36b-HOcs-LYS2* | This study |
| DDY3282 | S288C | *MAT***a***-inc ura3-52 lys2-801 ade2-101 ochre trp1-Δ63 his3-Δ200 leu2-Δ1::GAL1:HO-LEU2 rad52::HIS* *VII-L::URA3-TG36b-HOcs-LYS2 pif1-m2* | This study |
| DDY3283 | S288C | *MAT***a***-inc ura3-52 lys2-801 ade2-101 ochre trp1-Δ63 his3-Δ200 leu2-Δ1::GAL1:HO-LEU2 rad52::HIS* *VII-L::URA3-TG26c-HOcs-LYS2* | This study |
| DDY3284 | S288C | *MAT***a***-inc ura3-52 lys2-801 ade2-101 ochre trp1-Δ63 his3-Δ200 leu2-Δ1::GAL1:HO-LEU2 rad52::HIS* *VII-L::URA3-TG26c-HOcs-LYS2 pif1-m2* | This study |
| DDY3285 | S288C | *MAT***a***-inc ura3-52 lys2-801 ade2-101 ochre trp1-Δ63 his3-Δ200 leu2-Δ1::GAL1:HO-LEU2 rad52::HIS* *VII-L::URA3-TG36c-HOcs-LYS2* | This study |
| DDY3286 | S288C | *MAT***a***-inc ura3-52 lys2-801 ade2-101 ochre trp1-Δ63 his3-Δ200 leu2-Δ1::GAL1:HO-LEU2 rad52::HIS* *VII-L::URA3-TG36c-HOcs-LYS2 pif1-m2* | This study |
| MCY415 | BY4742 | *MAT*α *tlc1-tm::kanMX his3∆1 leu2∆0 ura3∆0* | This study |
| SSY76 | W303 | *MAT***a** *ade2-1 can1-100 leu2-3,112 his3-11,15 trp1-1 ura3-1 RAD5* *GALLp::natNT2-EST1 bar1∆LEU2 DIA5-1* (*ADE2* on the right arm of chromosome V) | This study |
| SSY292 | W303 | *MAT***a** *ade2-1 can1-100 leu2-3,112 his3-11,15 trp1-1 ura3-1 RAD5* *GALLp::natNT2-EST1 bar1∆LEU2 pif1-m2 DIA5-1* (*ADE2* on the right arm of chromosome V) | This study |
| DDY3042 | S288C | *MAT***a***-inc ura3-52 lys2-801 ade2-101 ochre trp1-Δ63 his3-Δ200 leu2-Δ1::GAL1:HO-LEU2 rad52::HIS* *VII-L-ADE2-TG82-HOcs-LYS2 tel1::KANMX* | This study |
| DDY3043 | S288C | *MAT***a***-inc ura3-52 lys2-801 ade2-101 ochre trp1-Δ63 his3-Δ200 leu2-Δ1::GAL1:HO-LEU2 rad52::HIS* *VII-L-ADE2-TG82-HOcs-LYS2 pif1-m2 tel1::KANMX* | This study |
| DDY3483 | S288C | *MAT***a***-inc ura3-52 lys2-801 ade2-101 ochre trp1-Δ63 his3-Δ200 leu2-Δ1::GAL1:HO-LEU2 rad52::HIS* *VII-L::URA3-TG18-HOcs-LYS2 tel1::KANMX* | This study |
| DDY3484 | S288C | *MAT***a***-inc ura3-52 lys2-801 ade2-101 ochre trp1-Δ63 his3-Δ200 leu2-Δ1::GAL1:HO-LEU2 rad52::HIS* *VII-L::URA3-TG18-HOcs-LYS2 pif1-m2 tel1::KANMX* | This study |
| DDY3485 | S288C | *MAT***a***-inc ura3-52 lys2-801 ade2-101 ochre trp1-Δ63 his3-Δ200 leu2-Δ1::GAL1:HO-LEU2 rad52::HIS* *VII-L::URA3-TG34-HOcs-LYS2 tel1::KANMX* | This study |
| DDY3486 | S288C | *MAT***a***-inc ura3-52 lys2-801 ade2-101 ochre trp1-Δ63 his3-Δ200 leu2-Δ1::GAL1:HO-LEU2 rad52::HIS* *VII-L::URA3-TG34-HOcs-LYS2 pif1-m2 tel1::KANMX* | This study |
| DDY3234 | S288C | *MAT***a***-inc ura3-52 lys2-801 ade2-101 ochre trp1-Δ63 his3-Δ200 leu2-Δ1::GAL1:HO-LEU2 rad52::HIS* *VII-L::URA3-TG34-HOcs-LYS2 pif1-m2 AUR1* | This study |
| DDY3236 | S288C | *MAT***a***-inc ura3-52 lys2-801 ade2-101 ochre trp1-Δ63 his3-Δ200 leu2-Δ1::GAL1:HO-LEU2 rad52::HIS* *VII-L::URA3-TG34-HOcs-LYS2 pif1-m2 AUR1::pif1-m1* | This study |
| DDY3244 | S288C | *MAT***a***-inc ura3-52 lys2-801 ade2-101 ochre trp1-Δ63 his3-Δ200 leu2-Δ1::GAL1:HO-LEU2 rad52::HIS* *VII-L::URA3-TG34-HOcs-LYS2 pif1-m2 AUR1::pif1-m1(5AQ)* | This study |
| DDY3224 | S288C | *MAT***a***-inc ura3-52 lys2-801 ade2-101 ochre trp1-Δ63 his3-Δ200 leu2-Δ1::GAL1:HO-LEU2 rad52::HIS* *VII-L::URA3-TG18-HOcs-LYS2 pif1-m2 AUR1* | This study |
| DDY3226 | S288C | *MAT***a***-inc ura3-52 lys2-801 ade2-101 ochre trp1-Δ63 his3-Δ200 leu2-Δ1::GAL1:HO-LEU2 rad52::HIS* *VII-L::URA3-TG18-HOcs-LYS2 pif1-m2 AUR1::pif1-m1* | This study |
| DDY3230 | S288C | *MAT***a***-inc ura3-52 lys2-801 ade2-101 ochre trp1-Δ63 his3-Δ200 leu2-Δ1::GAL1:HO-LEU2 rad52::HIS* *VII-L::URA3-TG18-HOcs-LYS2 pif1-m2 AUR1::pif1-m1(4A)* | This study |
| DDY3470 | S288C | *MAT***a***-inc ura3-52 lys2-801 ade2-101 ochre trp1-Δ63 his3-Δ200 leu2-Δ1::GAL1:HO-LEU2 rad52::HIS* *VII-L::URA3-TG18-HOcs-LYS2 pif1-m2 AUR1::pif1-m1(4D)* | This study |
| DDY3240 | S288C | *MAT***a***-inc ura3-52 lys2-801 ade2-101 ochre trp1-Δ63 his3-Δ200 leu2-Δ1::GAL1:HO-LEU2 rad52::HIS* *VII-L::URA3-TG34-HOcs-LYS2 pif1-m2 AUR1::pif1-m1(4D)* | This study |
| DDY3141 | S288C | *MAT***a***-inc ura3-52 lys2-801 ade2-101 ochre trp1-Δ63 his3-Δ200 leu2-Δ1::GAL1:HO-LEU2 rad52::HIS* *VII-L-ADE2-TG82-HOcs-LYS2 sml1::NATMX* | This study |
| DDY3142 | S288C | *MAT***a***-inc ura3-52 lys2-801 ade2-101 ochre trp1-Δ63 his3-Δ200 leu2-Δ1::GAL1:HO-LEU2 rad52::HIS* *VII-L-ADE2-TG82-HOcs-LYS2 pif1-m2 sml1::NATMX* | This study |
| DDY3144 | S288C | *MAT***a***-inc ura3-52 lys2-801 ade2-101 ochre trp1-Δ63 his3-Δ200 leu2-Δ1::GAL1:HO-LEU2 rad52::HIS* *VII-L-ADE2-TG82-HOcs-LYS2 sml1:NATMX: mec1::KANMX* | This study |
| DDY3039 | S288C | *MAT***a***-inc ura3-52 lys2-801 ade2-101 ochre trp1-Δ63 his3-Δ200 leu2-Δ1::GAL1:HO-LEU2 rad52::HIS* *VII-L-ADE2-TG82-HOcs-LYS2 pif1-m2 sml1:NATMX: mec1::KANMX* | This study |
| DDY3146 | S288C | *MAT***a***-inc ura3-52 lys2-801 ade2-101 ochre trp1-Δ63 his3-Δ200 leu2-Δ1::GAL1:HO-LEU2 rad52::HIS* *VII-L-ADE2-TG82-HOcs-LYS2 sml1::NATMX rad53::KANMX* | This study |
| DDY3041 | S288C | *MAT***a***-inc ura3-52 lys2-801 ade2-101 ochre trp1-Δ63 his3-Δ200 leu2-Δ1::GAL1:HO-LEU2 rad52::HIS* *VII-L-ADE2-TG82-HOcs-LYS2 pif1-m2 sml1::NATMX rad53::KANMX* | This study |
| DDY3224 | S288C | *MAT***a***-inc ura3-52 lys2-801 ade2-101 ochre trp1-Δ63 his3-Δ200 leu2-Δ1::GAL1:HO-LEU2 rad52::HIS* *VII-L::URA3-TG18-HOcs-LYS2 pif1-m2 AUR1* | This study |
| DDY3226 | S288C | *MAT***a***-inc ura3-52 lys2-801 ade2-101 ochre trp1-Δ63 his3-Δ200 leu2-Δ1::GAL1:HO-LEU2 rad52::HIS* *VII-L::URA3-TG18-HOcs-LYS2 pif1-m2 AUR1::pif1-m1* | This study |
| DDY3604 | S288C | *MAT***a***-inc ura3-52 lys2-801 ade2-101 ochre trp1-Δ63 his3-Δ200 leu2-Δ1::GAL1:HO-LEU2 rad52::HIS* *VII-L::TG18-HOcs-LYS2 ura3::HPHMX cdc13::KANMX* pRS414-Cdc13-Est1 | This study |
| DDY3605 | S288C | *MAT***a***-inc ura3-52 lys2-801 ade2-101 ochre trp1-Δ63 his3-Δ200 leu2-Δ1::GAL1:HO-LEU2 rad52::HIS* *VII-L::TG18-HOcs-LYS2 ura3::HPHMX cdc13::KANMX pif1-m2* pRS414-Cdc13-Est1 | This study |
| DDY3606 | S288C | *MAT***a***-inc ura3-52 lys2-801 ade2-101 ochre trp1-Δ63 his3-Δ200 leu2-Δ1::GAL1:HO-LEU2 rad52::HIS* *VII-L::TG34-HOcs-LYS2 ura3::HPHMX cdc13::KANMX* pRS414-Cdc13-Est1 | This study |
| DDY3607 | S288C | *MAT***a***-inc ura3-52 lys2-801 ade2-101 ochre trp1-Δ63 his3-Δ200 leu2-Δ1::GAL1:HO-LEU2 rad52::HIS* *VII-L::TG34-HOcs-LYS2 ura3::HPHMX cdc13::KANMX pif1-m2* pRS414-Cdc13-Est1 | This study |
| DDY3608 | S288C | *MAT***a***-inc ura3-52 lys2-801 ade2-101 ochre trp1-Δ63 his3-Δ200 leu2-Δ1::GAL1:HO-LEU2 rad52::HIS* *VII-L::TG18-HOcs-LYS2 ura3::HPHMX cdc13::KANMX* pRS414-Cdc13-Est2 | This study |
| DDY3609 | S288C | *MAT***a***-inc ura3-52 lys2-801 ade2-101 ochre trp1-Δ63 his3-Δ200 leu2-Δ1::GAL1:HO-LEU2 rad52::HIS* *VII-L::TG18-HOcs-LYS2 ura3::HPHMX cdc13::KANMX pif1-m2* pRS414-Cdc13-Est2 | This study |
| DDY3610 | S288C | *MAT***a***-inc ura3-52 lys2-801 ade2-101 ochre trp1-Δ63 his3-Δ200 leu2-Δ1::GAL1:HO-LEU2 rad52::HIS* *VII-L::TG34-HOcs-LYS2 ura3::HPHMX cdc13::KANMX* pRS414-Cdc13-Est2 | This study |
| DDY3611 | S288C | *MAT***a***-inc ura3-52 lys2-801 ade2-101 ochre trp1-Δ63 his3-Δ200 leu2-Δ1::GAL1:HO-LEU2 rad52::HIS* *VII-L::TG34-HOcs-LYS2 ura3::HPHMX cdc13::KANMX pif1-m2* pRS414-Cdc13-Est2 | This study |
| DDY3499 | S288C | *MAT***a***-inc ura3-52 lys2-801 ade2-101 ochre trp1-Δ63 his3-Δ200 leu2-Δ1::GAL1:HO-LEU2 rad52::HIS* *VII-L::URA3-TG18-HOcs-LYS2 est2-up34* | This study |
| DDY3500 | S288C | *MAT***a***-inc ura3-52 lys2-801 ade2-101 ochre trp1-Δ63 his3-Δ200 leu2-Δ1::GAL1:HO-LEU2 rad52::HIS* *VII-L::URA3-TG18-HOcs-LYS2 pif1-m2 est2up-34* | This study |
| DDY3501 | S288C | *MAT***a***-inc ura3-52 lys2-801 ade2-101 ochre trp1-Δ63 his3-Δ200 leu2-Δ1::GAL1:HO-LEU2 rad52::HIS* *VII-L::URA3-TG34-HOcs-LYS2 est2-up34* | This study |
| DDY3502 | S288C | *MAT***a***-inc ura3-52 lys2-801 ade2-101 ochre trp1-Δ63 his3-Δ200 leu2-Δ1::GAL1:HO-LEU2 rad52::HIS* *VII-L::URA3-TG34-HOcs-LYS2 pif1-m2 est2-up34* | This study |
| DDY3287 | S288C | *MAT***a***-inc ura3-52 lys2-801 ade2-101 ochre trp1-Δ63 his3-Δ200 leu2-Δ1::GAL1:HO-LEU2 rad52::HIS* *VII-L::URA3-TG26d-HOcs-LYS2* | This study |
| DDY3288 | S288C | *MAT***a***-inc ura3-52 lys2-801 ade2-101 ochre trp1-Δ63 his3-Δ200 leu2-Δ1::GAL1:HO-LEU2 rad52::HIS* *VII-L::URA3-TG26d-HOcs-LYS2 pif1-m2* | This study |
| DDY3289 | S288C | *MAT***a***-inc ura3-52 lys2-801 ade2-101 ochre trp1-Δ63 his3-Δ200 leu2-Δ1::GAL1:HO-LEU2 rad52::HIS* *VII-L::URA3-TG36d-HOcs-LYS2* | This study |
| DDY3290 | S288C | *MAT***a***-inc ura3-52 lys2-801 ade2-101 ochre trp1-Δ63 his3-Δ200 leu2-Δ1::GAL1:HO-LEU2 rad52::HIS* *VII-L::URA3-TG36d-HOcs-LYS2 pif1-m2* | This study |
| DDY3291 | S288C | *MAT***a***-inc ura3-52 lys2-801 ade2-101 ochre trp1-Δ63 his3-Δ200 leu2-Δ1::GAL1:HO-LEU2 rad52::HIS* *VII-L::URA3-TG26e-HOcs-LYS2* | This study |
| DDY3292 | S288C | *MAT***a***-inc ura3-52 lys2-801 ade2-101 ochre trp1-Δ63 his3-Δ200 leu2-Δ1::GAL1:HO-LEU2 rad52::HIS* *VII-L::URA3-TG26e-HOcs-LYS2 pif1-m2* | This study |
| DDY3293 | S288C | *MAT***a***-inc ura3-52 lys2-801 ade2-101 ochre trp1-Δ63 his3-Δ200 leu2-Δ1::GAL1:HO-LEU2 rad52::HIS* *VII-L::URA3-TG36e-HOcs-LYS2* | This study |
| DDY3294 | S288C | *MAT***a***-inc ura3-52 lys2-801 ade2-101 ochre trp1-Δ63 his3-Δ200 leu2-Δ1::GAL1:HO-LEU2 rad52::HIS* *VII-L::URA3-TG36e-HOcs-LYS2 pif1-m2* | This study |
| DDY3324 | S288C | *MAT***a***-inc ura3-52 lys2-801 ade2-101 ochre trp1-Δ63 his3-Δ200 leu2-Δ1::GAL1:HO-LEU2 rad52::HIS* *VII-L::URA3-Rap1x0+TG14-HOcs-LYS2* | This study |
| DDY3325 | S288C | *MAT***a***-inc ura3-52 lys2-801 ade2-101 ochre trp1-Δ63 his3-Δ200 leu2-Δ1::GAL1:HO-LEU2 rad52::HIS* *VII-L::URA3-Rap1x0+TG14-HOcs-LYS2 pif1-m2* | This study |
| DDY3326 | S288C | *MAT***a***-inc ura3-52 lys2-801 ade2-101 ochre trp1-Δ63 his3-Δ200 leu2-Δ1::GAL1:HO-LEU2 rad52::HIS* *VII-L::URA3-Rap1x1+TG14-HOcs-LYS2* | This study |
| DDY3327 | S288C | *MAT***a***-inc ura3-52 lys2-801 ade2-101 ochre trp1-Δ63 his3-Δ200 leu2-Δ1::GAL1:HO-LEU2 rad52::HIS* *VII-L::URA3-Rap1x1+TG14-HOcs-LYS2 pif1-m2* | This study |
| DDY3328 | S288C | *MAT***a***-inc ura3-52 lys2-801 ade2-101 ochre trp1-Δ63 his3-Δ200 leu2-Δ1::GAL1:HO-LEU2 rad52::HIS* *VII-L::URA3-Rap1x2+TG14-HOcs-LYS2* | This study |
| DDY3329 | S288C | *MAT***a***-inc ura3-52 lys2-801 ade2-101 ochre trp1-Δ63 his3-Δ200 leu2-Δ1::GAL1:HO-LEU2 rad52::HIS* *VII-L::URA3-Rap1x2+TG14-HOcs-LYS2 pif1-m2* | This study |
| DDY3330 | S288C | *MAT***a***-inc ura3-52 lys2-801 ade2-101 ochre trp1-Δ63 his3-Δ200 leu2-Δ1::GAL1:HO-LEU2 rad52::HIS* *VII-L::URA3-Rap1x3+TG14-HOcs-LYS2* | This study |
| DDY3331 | S288C | *MAT***a***-inc ura3-52 lys2-801 ade2-101 ochre trp1-Δ63 his3-Δ200 leu2-Δ1::GAL1:HO-LEU2 rad52::HIS* *VII-L::URA3-Rap1x3+TG14-HOcs-LYS2 pif1-m2* | This study |
| DDY3332 | S288C | *MAT***a***-inc ura3-52 lys2-801 ade2-101 ochre trp1-Δ63 his3-Δ200 leu2-Δ1::GAL1:HO-LEU2 rad52::HIS* *VII-L::URA3-Rap1x4+TG14-HOcs-LYS2* | This study |
| DDY3333 | S288C | *MAT***a***-inc ura3-52 lys2-801 ade2-101 ochre trp1-Δ63 his3-Δ200 leu2-Δ1::GAL1:HO-LEU2 rad52::HIS* *VII-L::URA3-Rap1x4+TG14-HOcs-LYS2 pif1-m2* | This study |
| DDY3334 | S288C | *MAT***a***-inc ura3-52 lys2-801 ade2-101 ochre trp1-Δ63 his3-Δ200 leu2-Δ1::GAL1:HO-LEU2 rad52::HIS* *VII-L::URA3-Rap1x0-HOcs-LYS2* | This study |
| DDY3335 | S288C | *MAT***a***-inc ura3-52 lys2-801 ade2-101 ochre trp1-Δ63 his3-Δ200 leu2-Δ1::GAL1:HO-LEU2 rad52::HIS* *VII-L::URA3-Rap1x0-HOcs-LYS2 pif1-m2* | This study |
| DDY3336 | S288C | *MAT***a***-inc ura3-52 lys2-801 ade2-101 ochre trp1-Δ63 his3-Δ200 leu2-Δ1::GAL1:HO-LEU2 rad52::HIS* *VII-L::URA3-Rap1x1-HOcs-LYS2* | This study |
| DDY3337 | S288C | *MAT***a***-inc ura3-52 lys2-801 ade2-101 ochre trp1-Δ63 his3-Δ200 leu2-Δ1::GAL1:HO-LEU2 rad52::HIS* *VII-L::URA3-Rap1x1-HOcs-LYS2 pif1-m2* | This study |
| DDY3338 | S288C | *MAT***a***-inc ura3-52 lys2-801 ade2-101 ochre trp1-Δ63 his3-Δ200 leu2-Δ1::GAL1:HO-LEU2 rad52::HIS* *VII-L::URA3-Rap1x2-HOcs-LYS2* | This study |
| DDY3339 | S288C | *MAT***a***-inc ura3-52 lys2-801 ade2-101 ochre trp1-Δ63 his3-Δ200 leu2-Δ1::GAL1:HO-LEU2 rad52::HIS* *VII-L::URA3-Rap1x2-HOcs-LYS2 pif1-m2* | This study |
| DDY3340 | S288C | *MAT***a***-inc ura3-52 lys2-801 ade2-101 ochre trp1-Δ63 his3-Δ200 leu2-Δ1::GAL1:HO-LEU2 rad52::HIS* *VII-L::URA3-Rap1x3-HOcs-LYS2* | This study |
| DDY3341 | S288C | *MAT***a***-inc ura3-52 lys2-801 ade2-101 ochre trp1-Δ63 his3-Δ200 leu2-Δ1::GAL1:HO-LEU2 rad52::HIS* *VII-L::URA3-Rap1x3-HOcs-LYS2 pif1-m2* | This study |
| DDY3342 | S288C | *MAT***a***-inc ura3-52 lys2-801 ade2-101 ochre trp1-Δ63 his3-Δ200 leu2-Δ1::GAL1:HO-LEU2 rad52::HIS* *VII-L::URA3-Rap1x4-HOcs-LYS2* | This study |
| DDY3343 | S288C | *MAT***a***-inc ura3-52 lys2-801 ade2-101 ochre trp1-Δ63 his3-Δ200 leu2-Δ1::GAL1:HO-LEU2 rad52::HIS* *VII-L::URA3-Rap1x4-HOcs-LYS2 pif1-m2* | This study |
| DDY3475 | S288C | *MAT***a***-inc ura3-52 lys2-801 ade2-101 ochre trp1-Δ63 his3-Δ200 leu2-Δ1::GAL1:HO-LEU2 rad52::HIS* *VII-L::URA3-TG18-HOcs-LYS2 rif1::KANMX* | This study |
| DDY3476 | S288C | *MAT***a***-inc ura3-52 lys2-801 ade2-101 ochre trp1-Δ63 his3-Δ200 leu2-Δ1::GAL1:HO-LEU2 rad52::HIS* *VII-L::URA3-TG18-HOcs-LYS2 pif1-m2 rif1::KANMX* | This study |
| DDY3477 | S288C | *MAT***a***-inc ura3-52 lys2-801 ade2-101 ochre trp1-Δ63 his3-Δ200 leu2-Δ1::GAL1:HO-LEU2 rad52::HIS* *VII-L::URA3-TG34-HOcs-LYS2 rif1::KANMX* | This study |
| DDY3478 | S288C | *MAT***a***-inc ura3-52 lys2-801 ade2-101 ochre trp1-Δ63 his3-Δ200 leu2-Δ1::GAL1:HO-LEU2 rad52::HIS* *VII-L::URA3-TG34-HOcs-LYS2 pif1-m2 rif1::KANMX* | This study |
| DDY3479 | S288C | *MAT***a***-inc ura3-52 lys2-801 ade2-101 ochre trp1-Δ63 his3-Δ200 leu2-Δ1::GAL1:HO-LEU2 rad52::HIS* *VII-L::URA3-TG18-HOcs-LYS2 rif2::NATMX* | This study |
| DDY3480 | S288C | *MAT***a***-inc ura3-52 lys2-801 ade2-101 ochre trp1-Δ63 his3-Δ200 leu2-Δ1::GAL1:HO-LEU2 rad52::HIS* *VII-L::URA3-TG18-HOcs-LYS2 pif1-m2 rif2::NATMX* | This study |
| DDY3481 | S288C | *MAT***a***-inc ura3-52 lys2-801 ade2-101 ochre trp1-Δ63 his3-Δ200 leu2-Δ1::GAL1:HO-LEU2 rad52::HIS* *VII-L::URA3-TG34-HOcs-LYS2 rif2::NATMX* | This study |
| DDY3482 | S288C | *MAT***a***-inc ura3-52 lys2-801 ade2-101 ochre trp1-Δ63 his3-Δ200 leu2-Δ1::GAL1:HO-LEU2 rad52::HIS* *VII-L::URA3-TG34-HOcs-LYS2 pif1-m2 rif2::NATMX* | This study |
| DDY3466 | S288C | *MAT***a***-inc ura3-52 lys2-801 ade2-101 ochre trp1-Δ63 his3-Δ200 leu2-Δ1::GAL1:HO-LEU2 rad52::HIS* *VII-L::URA3-TG18-HOcs-LYS2 rif1::KANMX rif2::NATMX* | This study |
| DDY3467 | S288C | *MAT***a***-inc ura3-52 lys2-801 ade2-101 ochre trp1-Δ63 his3-Δ200 leu2-Δ1::GAL1:HO-LEU2 rad52::HIS* *VII-L::URA3-TG18-HOcs-LYS2 pif1-m2 rif1::KANMX rif2::NATMX* | This study |
| DDY3468 | S288C | *MAT***a***-inc ura3-52 lys2-801 ade2-101 ochre trp1-Δ63 his3-Δ200 leu2-Δ1::GAL1:HO-LEU2 rad52::HIS* *VII-L::URA3-TG34-HOcs-LYS2 rif1::KANMX rif2::NATMX* | This study |
| DDY3469 | S288C | *MAT***a***-inc ura3-52 lys2-801 ade2-101 ochre trp1-Δ63 his3-Δ200 leu2-Δ1::GAL1:HO-LEU2 rad52::HIS* *VII-L::URA3-TG34-HOcs-LYS2 pif1-m2 rif1::KANMX rif2::NATMX* | This study |
| DDY3526 | S288C | *MAT***a***-inc ura3-52 lys2-801 ade2-101 ochre trp1-Δ63 his3-Δ200 leu2-Δ1::GAL1:HO-LEU2 rad52::HIS* *VII-L::TG18-HOcs-LYS2 ura3::HPHMX cdc13::KANMX* YEp-*URA3-CDC13* | This study |
| DDY3527 | S288C | *MAT***a***-inc ura3-52 lys2-801 ade2-101 ochre trp1-Δ63 his3-Δ200 leu2-Δ1::GAL1:HO-LEU2 rad52::HIS* *VII-L::URA3-TG18-HOcs-LYS2 pif1-m2 ura3::HPHMX cdc13::KANMX* YEp-*URA3-CDC13* | This study |
| DDY3528 | S288C | *MAT***a***-inc ura3-52 lys2-801 ade2-101 ochre trp1-Δ63 his3-Δ200 leu2-Δ1::GAL1:HO-LEU2 rad52::HIS* *VII-L::TG18-HOcs-LYS2 ura3::HPHMX cdc13::KANMX* YEp-*URA3-CDC13* | This study |
| DDY3530 | S288C | *MAT***a***-inc ura3-52 lys2-801 ade2-101 ochre trp1-Δ63 his3-Δ200 leu2-Δ1::GAL1:HO-LEU2 rad52::HIS* *VII-L::URA3-TG18-HOcs-LYS2 pif1-m2 ura3::HPHMX cdc13::KANMX* YEp-*URA3-CDC13* | This study |
| DDY3589 | S288C | *MAT***a***-inc ura3-52 lys2-801 ade2-101 ochre trp1-Δ63 his3-Δ200 leu2-Δ1::GAL1:HO rad52::HIS* *VII-L::TG18-HOcs-LYS2 leu2::NAT ura3::HPHMX cdc13::KANMX* YEp-*URA3-CDC13 pRAD52-TRP* | This study |
| DDY3591 | S288C | *MAT***a***-inc ura3-52 lys2-801 ade2-101 ochre trp1-Δ63 his3-Δ200 leu2-Δ1::GAL1:HO rad52::HIS* *VII-L::URA3-TG18-HOcs-LYS2 pif1-m2 leu2::NAT ura3::HPHMX cdc13::KANMX* YEp-*URA3-CDC13 pRAD52-TRP* | This study |
| DDY3534 | S288C | *MAT***a***-inc ura3-52 lys2-801 ade2-101 ochre trp1-Δ63 his3-Δ200 leu2-Δ1::GAL1:HO-LEU2 rad52::HIS* *VII-L::TG18-HOcs-LYS2 ura3::HPHMX cdc13::KANMX* pRS414-*CDC13* | This study |
| DDY3536 | S288C | *MAT***a***-inc ura3-52 lys2-801 ade2-101 ochre trp1-Δ63 his3-Δ200 leu2-Δ1::GAL1:HO-LEU2 rad52::HIS* *VII-L::TG18-HOcs-LYS2 ura3::HPHMX cdc13::KANMX pif1-m2* pRS414-*CDC13* | This study |
| DDY3535 | S288C | *MAT***a***-inc ura3-52 lys2-801 ade2-101 ochre trp1-Δ63 his3-Δ200 leu2-Δ1::GAL1:HO-LEU2 rad52::HIS* *VII-L::TG18-HOcs-LYS2 ura3::HPHMX cdc13::KANMX* pRS414-*cdc13-L91A* | This study |
| DDY3537 | S288C | *MAT***a***-inc ura3-52 lys2-801 ade2-101 ochre trp1-Δ63 his3-Δ200 leu2-Δ1::GAL1:HO-LEU2 rad52::HIS* *VII-L::TG18-HOcs-LYS2 ura3::HPHMX cdc13::KANMX pif1-m2* pRS414-*cdc13-L91A* | This study |
| DDY3538 | S288C | *MAT***a***-inc ura3-52 lys2-801 ade2-101 ochre trp1-Δ63 his3-Δ200 leu2-Δ1::GAL1:HO-LEU2 rad52::HIS* *VII-L::TG34-HOcs-LYS2 ura3::HPHMX cdc13::KANMX* pRS414-*CDC13* | This study |
| DDY3540 | S288C | *MAT***a***-inc ura3-52 lys2-801 ade2-101 ochre trp1-Δ63 his3-Δ200 leu2-Δ1::GAL1:HO-LEU2 rad52::HIS* *VII-L::TG34-HOcs-LYS2 ura3::HPHMX cdc13::KANMX pif1-m2* pRS414-*CDC13* | This study |
| DDY3539 | S288C | *MAT***a***-inc ura3-52 lys2-801 ade2-101 ochre trp1-Δ63 his3-Δ200 leu2-Δ1::GAL1:HO-LEU2 rad52::HIS* *VII-L::TG34-HOcs-LYS2 ura3::HPHMX cdc13::KANMX* pRS414-*cdc13-L91A* | This study |
| DDY3541 | S288C | *MAT***a***-inc ura3-52 lys2-801 ade2-101 ochre trp1-Δ63 his3-Δ200 leu2-Δ1::GAL1:HO-LEU2 rad52::HIS* *VII-L::TG34-HOcs-LYS2 ura3::HPHMX cdc13::KANMX pif1-m2* pRS414-*cdc13-L91A* | This study |
| DDY3520 | S288C | *MAT***a***-inc ura3-52 lys2-801 ade2-101 ochre trp1-Δ63 his3-Δ200 leu2-Δ1::GAL1:HO-LEU2 rad52::HIS* *VII-L::TG34-HOcs-LYS2 ura3::HPH cdc13::KANMX YEP24-CDC13 pRAD52* | This study |
| DDY3522 | S288C | *MAT***a***-inc ura3-52 lys2-801 ade2-101 ochre trp1-Δ63 his3-Δ200 leu2-Δ1::GAL1:HO-LEU2 rad52::HIS* *VII-L::TG34-HOcs-LYS2 ura3::HPH cdc13::KANMX YEP24-CDC13 pRAD52 pif1-m2* | This study |
| DDY3528 | S288C | *MAT***a***-inc ura3-52 lys2-801 ade2-101 ochre trp1-Δ63 his3-Δ200 leu2-Δ1::GAL1:HO-LEU2 rad52::HIS* *VII-L::TG34-HOcs-LYS2 ura3::HPH cdc13::KANMX YEP24-CDC13* | This study |
| DDY3530 | S288C | *MAT***a***-inc ura3-52 lys2-801 ade2-101 ochre trp1-Δ63 his3-Δ200 leu2-Δ1::GAL1:HO-LEU2 rad52::HIS* *VII-L::TG34-HOcs-LYS2 ura3::HPH cdc13::KANMX YEP24-CDC13 pif1-m2* | This study |
| DDY3584 | S288C | *MAT***a***-inc ura3-52 lys2-801 ade2-101 ochre trp1-Δ63 his3-Δ200 leu2-Δ1::GAL1:HO-LEU2 rad52::HIS* *VII-L::TG34-HOcs-LYS2 ura3::HPH leu2::NAT cdc13::KANMX YEP24-CDC13* | This study |
| DDY3586 | S288C | *MAT***a***-inc ura3-52 lys2-801 ade2-101 ochre trp1-Δ63 his3-Δ200 leu2-Δ1::GAL1:HO-LEU2 rad52::HIS* *VII-L::TG34-HOcs-LYS2 ura3::HPH leu2::NAT cdc13::KANMX YEP24-CDC13 pif1-m2* | This study |
| DDY3589 | S288C | *MAT***a***-inc ura3-52 lys2-801 ade2-101 ochre trp1-Δ63 his3-Δ200 leu2-Δ1::GAL1:HO-LEU2 rad52::HIS* *VII-L::TG34-HOcs-LYS2 ura3::HPH leu2::NAT cdc13::KANMX YEP24-CDC13 pRAD52* | This study |
| DDY3591 | S288C | *MAT***a***-inc ura3-52 lys2-801 ade2-101 ochre trp1-Δ63 his3-Δ200 leu2-Δ1::GAL1:HO-LEU2 rad52::HIS* *VII-L::TG34-HOcs-LYS2 ura3::HPH leu2::NAT cdc13::KANMX YEP24-CDC13 pRAD52 pif1-m2* | This study |
| DDY3248 | S288C | *MAT***a***-inc ura3-52 lys2-801 ade2-101 ochre trp1-Δ63 his3-Δ200 leu2-Δ1::GAL1:HO-LEU2 rad52::HIS* *VII-L::URA3-TG96-HOcs-LYS2* | This study |
| DDY3250 | S288C | *MAT***a***-inc ura3-52 lys2-801 ade2-101 ochre trp1-Δ63 his3-Δ200 leu2-Δ1::GAL1:HO-LEU2 rad52::HIS* *VII-L::URA3-TG119-HOcs-LYS2* | This study |
| DDY3252 | S288C | *MAT***a***-inc ura3-52 lys2-801 ade2-101 ochre trp1-Δ63 his3-Δ200 leu2-Δ1::GAL1:HO-LEU2 rad52::HIS* *VII-L::URA3-TG142-HOcs-LYS2* | This study |
| DDY3133 | S288C | *MAT***a***-inc ura3-52 lys2-801 ade2-101 ochre trp1-Δ63 his3-Δ200 leu2-Δ1::GAL1:HO-LEU2 rad52::HIS* *VII-L::URA3-TG162-HOcs-LYS2* | This study |
| DDY3134 | S288C | *MAT***a***-inc ura3-52 lys2-801 ade2-101 ochre trp1-Δ63 his3-Δ200 leu2-Δ1::GAL1:HO-LEU2 rad52::HIS* *VII-L::URA3-TG162-HOcs-LYS2 pif1-m2* | This study |
| DDY3582 | S288C | *MAT***a***-inc ura3-52 lys2-801 ade2-101 ochre trp1-Δ63 his3-Δ200 leu2-Δ1::GAL1:HO-LEU2 rad52::HIS* *VII-L::TG18-HOcs-LYS2 ura3::HPH cdc13::KAN YEP24-CDC13* | This study |
| DDY3583 | S288C | *MAT***a***-inc ura3-52 lys2-801 ade2-101 ochre trp1-Δ63 his3-Δ200 leu2-Δ1::GAL1:HO-LEU2 rad52::HIS* *VII-L::TG18-HOcs-LYS2 ura3::HPH cdc13::KAN YEP24-CDC13 pif1-m2* | This study |
| DDY3529 | S288C | *MAT***a***-inc ura3-52 lys2-801 ade2-101 ochre trp1-Δ63 his3-Δ200 leu2-Δ1::GAL1:HO-LEU2 rad52::HIS* *VII-L::TG34-HOcs-LYS2 ura3::HPH cdc13::KAN YEP24-CDC13* | This study |
| DDY3614 | S288C | DDY3529 *leu2::NATMX* | This study |
| DDY3531 | S288C | *MAT***a***-inc ura3-52 lys2-801 ade2-101 ochre trp1-Δ63 his3-Δ200 leu2-Δ1::GAL1:HO-LEU2 rad52::HIS* *VII-L::TG34-HOcs-LYS2 ura3::HPH cdc13::KAN YEP24-CDC13 pif1-m2* | This study |
| DDY3615 | S288C | DDY3531 *leu2::NATMX* | This study |
| DDY3768 | S288C | DDY3614 pRS425-*CDC13* | This study |
| DDY3769 | S288C | DDY3614 pRS425-*cdc13-L91A* | This study |
| DDY3770 | S288C | DDY3614 pRS425-*cdc13-F236S* | This study |
| DDY3771 | S288C | DDY3614 pRS425-*cdc13-Q256H* | This study |
| DDY3772 | S288C | DDY3614 pRS425-*cdc13-Q583K* | This study |
| DDY3773 | S288C | DDY3614 pRS425-*cdc13-I87N* | This study |
| DDY3774 | S288C | DDY3614 pRS425-*cdc13-Y758N* | This study |
| DDY3775 | S288C | DDY3614 pRS425-*cdc13-H12R* | This study |
| DDY3776 | S288C | DDY3614 pRS425-*cdc13-E556V/N567D* | This study |
| DDY3777 | S288C | DDY3614 pRS425-*cdc13-F728I* | This study |
| DDY3778 | S288C | DDY3614 pRS425-*cdc13-E252K* | This study |
| DDY3779 | S288C | DDY3614 pRS425-*cdc13-P235S* | This study |
| DDY3780 | S288C | DDY3614 pRS425-*cdc13-S255A* | This study |
| DDY3781 | S288C | DDY3614 pRS425-*cdc13-K50Q* | This study |
| DDY3782 | S288C | DDY3614 pRS425-*cdc13-F237V* | This study |
| DDY3783 | S288C | DDY3615 pRS425-*CDC13* | This study |
| DDY3784 | S288C | DDY3615 pRS425-*cdc13-L91A* | This study |
| DDY3785 | S288C | DDY3615 pRS425-*cdc13-F236S* | This study |
| DDY3786 | S288C | DDY3615 pRS425-*cdc13-Q256H* | This study |
| DDY3787 | S288C | DDY3615 pRS425-*cdc13-Q583K* | This study |
| DDY3788 | S288C | DDY3615 pRS425-*cdc13-I87N* | This study |
| DDY3789 | S288C | DDY3615 pRS425-*cdc13-Y758N* | This study |
| DDY3790 | S288C | DDY3615 pRS425-*cdc13-H12R* | This study |
| DDY3791 | S288C | DDY3615 pRS425-*cdc13-E556V/N567D* | This study |
| DDY3792 | S288C | DDY3615 pRS425-*cdc13-F728I* | This study |
| DDY3793 | S288C | DDY3615 pRS425-*cdc13-E252K* | This study |
| DDY3794 | S288C | DDY3615 pRS425-*cdc13-P235S* | This study |
| DDY3795 | S288C | DDY3615 pRS425-*cdc13-S255A* | This study |
| DDY3796 | S288C | DDY3615 pRS425-*cdc13-K50Q* | This study |
| DDY3797 | S288C | DDY3615 pRS425-*cdc13-F237V* | This study |
| DDY4722 | S288C | DDY3582 pRS415-*CDC13* | This study |
| DDY4723 | S288C | DDY3582 pRS415-*cdc13-L91A* | This study |
| DDY4724 | S288C | DDY3582 pRS425-*CDC13* | This study |
| DDY4725 | S288C | DDY3582 pRS425-*cdc13-L91A* | This study |
| DDY4726 | S288C | DDY3583 pRS415-*CDC13* | This study |
| DDY4727 | S288C | DDY3583 pRS415-*cdc13-L91A* | This study |
| DDY4728 | S288C | DDY3583 pRS425-*CDC13* | This study |
| DDY4729 | S288C | DDY3583 pRS425-*cdc13-L91A* | This study |
| DDY4730 | S288C | DDY3614 pRS415-*CDC13* | This study |
| DDY4731 | S288C | DDY3614 pRS415-*cdc13-L91A* | This study |
| DDY4732 | S288C | DDY3615 pRS415-*CDC13* | This study |
| DDY4733 | S288C | DDY3615 pRS415-*cdc13-L91A* | This study |
| DDY4588 | S288C | *MAT***a***-inc ura3-52 lys2-801 ade2-101 ochre trp1-Δ63 his3-Δ200 leu2-Δ1::GAL1:HO-LEU2 rad52::HIS* *VII-L::TG18-HOcs-LYS2 ura3::HPH cdc13::KAN pRS425-CDC13* | This study |
| DDY4589 | S288C | *MAT***a***-inc ura3-52 lys2-801 ade2-101 ochre trp1-Δ63 his3-Δ200 leu2-Δ1::GAL1:HO-LEU2 rad52::HIS* *VII-L::TG18-HOcs-LYS2 ura3::HPH cdc13::KAN pRS425-CDC13* | This study |
| DDY4590 | S288C | *MAT***a***-inc ura3-52 lys2-801 ade2-101 ochre trp1-Δ63 his3-Δ200 leu2-Δ1::GAL1:HO-LEU2 rad52::HIS* *VII-L::TG34-HOcs-LYS2 ura3::HPH cdc13::KAN pRS425-CDC13* | This study |
| DDY4591 | S288C | *MAT***a***-inc ura3-52 lys2-801 ade2-101 ochre trp1-Δ63 his3-Δ200 leu2-Δ1::GAL1:HO-LEU2 rad52::HIS* *VII-L::TG34-HOcs-LYS2 ura3::HPH cdc13::KAN pRS425-CDC13* | This study |
| DDY4592 | S288C | *MAT***a***-inc ura3-52 lys2-801 ade2-101 ochre trp1-Δ63 his3-Δ200 leu2-Δ1::GAL1:HO-LEU2 rad52::HIS* *VII-L::TG42-HOcs-LYS2 ura3::HPH cdc13::KAN pRS425-CDC13* | This study |
| DDY4593 | S288C | *MAT***a***-inc ura3-52 lys2-801 ade2-101 ochre trp1-Δ63 his3-Δ200 leu2-Δ1::GAL1:HO-LEU2 rad52::HIS* *VII-L::TG42-HOcs-LYS2 ura3::HPH cdc13::KAN pRS425-CDC13* | This study |
| DDY4594 | S288C | *MAT***a***-inc ura3-52 lys2-801 ade2-101 ochre trp1-Δ63 his3-Δ200 leu2-Δ1::GAL1:HO-LEU2 rad52::HIS* *VII-L::TG50-HOcs-LYS2 ura3::HPH cdc13::KAN pRS425-CDC13* | This study |
| DDY4595 | S288C | *MAT***a***-inc ura3-52 lys2-801 ade2-101 ochre trp1-Δ63 his3-Δ200 leu2-Δ1::GAL1:HO-LEU2 rad52::HIS* *VII-L::TG50-HOcs-LYS2 ura3::HPH cdc13::KAN pRS425-CDC13* | This study |
| DDY4596 | S288C | *MAT***a***-inc ura3-52 lys2-801 ade2-101 ochre trp1-Δ63 his3-Δ200 leu2-Δ1::GAL1:HO-LEU2 rad52::HIS* *VII-L::TG58-HOcs-LYS2 ura3::HPH cdc13::KAN pRS425-CDC13* | This study |
| DDY4597 | S288C | *MAT***a***-inc ura3-52 lys2-801 ade2-101 ochre trp1-Δ63 his3-Δ200 leu2-Δ1::GAL1:HO-LEU2 rad52::HIS* *VII-L::TG58-HOcs-LYS2 ura3::HPH cdc13::KAN pRS425-CDC13* | This study |
| DDY4598 | S288C | *MAT***a***-inc ura3-52 lys2-801 ade2-101 ochre trp1-Δ63 his3-Δ200 leu2-Δ1::GAL1:HO-LEU2 rad52::HIS* *VII-L::TG66-HOcs-LYS2 ura3::HPH cdc13::KAN pRS425-CDC13* | This study |
| DDY4600 | S288C | *MAT***a***-inc ura3-52 lys2-801 ade2-101 ochre trp1-Δ63 his3-Δ200 leu2-Δ1::GAL1:HO-LEU2 rad52::HIS* *VII-L::TG82-HOcs-LYS2 ura3::HPH cdc13::KAN pRS425-CDC13* | This study |
| DDY4601 | S288C | *MAT***a***-inc ura3-52 lys2-801 ade2-101 ochre trp1-Δ63 his3-Δ200 leu2-Δ1::GAL1:HO-LEU2 rad52::HIS* *VII-L::TG82-HOcs-LYS2 ura3::HPH cdc13::KAN pRS425-CDC13* | This study |
| DDY4604 | S288C | *MAT***a***-inc ura3-52 lys2-801 ade2-101 ochre trp1-Δ63 his3-Δ200 leu2-Δ1::GAL1:HO-LEU2 rad52::HIS* *VII-L::TG34-HOcs-LYS2 ura3::HPH cdc13::KAN pif1-m2 pRS425-CDC13* | This study |
| DDY4609 | S288C | *MAT***a***-inc ura3-52 lys2-801 ade2-101 ochre trp1-Δ63 his3-Δ200 leu2-Δ1::GAL1:HO-LEU2 rad52::HIS* *VII-L::TG58-HOcs-LYS2 ura3::HPH cdc13::KAN pif1-m2 pRS425-CDC13* | This study |
| DDY4610 | S288C | *MAT***a***-inc ura3-52 lys2-801 ade2-101 ochre trp1-Δ63 his3-Δ200 leu2-Δ1::GAL1:HO-LEU2 rad52::HIS* *VII-L::TG58-HOcs-LYS2 ura3::HPH cdc13::KAN pif1-m2 pRS425-CDC13* | This study |
| DDY4611 | S288C | *MAT***a***-inc ura3-52 lys2-801 ade2-101 ochre trp1-Δ63 his3-Δ200 leu2-Δ1::GAL1:HO-LEU2 rad52::HIS* *VII-L::TG66-HOcs-LYS2 ura3::HPH cdc13::KAN pif1-m2 pRS425-CDC13* | This study |
| DDY4612 | S288C | *MAT***a***-inc ura3-52 lys2-801 ade2-101 ochre trp1-Δ63 his3-Δ200 leu2-Δ1::GAL1:HO-LEU2 rad52::HIS* *VII-L::TG66-HOcs-LYS2 ura3::HPH cdc13::KAN pif1-m2 pRS425-CDC13* | This study |
| DDY4613 | S288C | *MAT***a***-inc ura3-52 lys2-801 ade2-101 ochre trp1-Δ63 his3-Δ200 leu2-Δ1::GAL1:HO-LEU2 rad52::HIS* *VII-L::TG74-HOcs-LYS2 ura3::HPH cdc13::KAN pif1-m2 pRS425-CDC13* | This study |
| DDY4614 | S288C | *MAT***a***-inc ura3-52 lys2-801 ade2-101 ochre trp1-Δ63 his3-Δ200 leu2-Δ1::GAL1:HO-LEU2 rad52::HIS* *VII-L::TG74-HOcs-LYS2 ura3::HPH cdc13::KAN pif1-m2 pRS425-CDC13* | This study |
| DDY4618 | S288C | *MAT***a***-inc ura3-52 lys2-801 ade2-101 ochre trp1-Δ63 his3-Δ200 leu2-Δ1::GAL1:HO-LEU2 rad52::HIS* *VII-L::TG18-HOcs-LYS2 ura3::HPH cdc13::KAN pRS425-cdc13-Q256H* | This study |
| DDY4619 | S288C | *MAT***a***-inc ura3-52 lys2-801 ade2-101 ochre trp1-Δ63 his3-Δ200 leu2-Δ1::GAL1:HO-LEU2 rad52::HIS* *VII-L::TG18-HOcs-LYS2 ura3::HPH cdc13::KAN pRS425-cdc13-Q256H* | This study |
| DDY4620 | S288C | *MAT***a***-inc ura3-52 lys2-801 ade2-101 ochre trp1-Δ63 his3-Δ200 leu2-Δ1::GAL1:HO-LEU2 rad52::HIS* *VII-L::TG74-HOcs-LYS2 ura3::HPH cdc13::KAN pRS425-CDC13* | This study |
| DDY4621 | S288C | *MAT***a***-inc ura3-52 lys2-801 ade2-101 ochre trp1-Δ63 his3-Δ200 leu2-Δ1::GAL1:HO-LEU2 rad52::HIS* *VII-L::TG74-HOcs-LYS2 ura3::HPH cdc13::KAN pRS425-CDC13* | This study |
| DDY4622 | S288C | *MAT***a***-inc ura3-52 lys2-801 ade2-101 ochre trp1-Δ63 his3-Δ200 leu2-Δ1::GAL1:HO-LEU2 rad52::HIS* *VII-L::TG18-HOcs-LYS2 ura3::HPH cdc13::KAN pif1-m2 pRS425-CDC13* | This study |
| DDY4623 | S288C | *MAT***a***-inc ura3-52 lys2-801 ade2-101 ochre trp1-Δ63 his3-Δ200 leu2-Δ1::GAL1:HO-LEU2 rad52::HIS* *VII-L::TG18-HOcs-LYS2 ura3::HPH cdc13::KAN pif1-m2 pRS425-CDC13* | This study |
| DDY4624 | S288C | *MAT***a***-inc ura3-52 lys2-801 ade2-101 ochre trp1-Δ63 his3-Δ200 leu2-Δ1::GAL1:HO-LEU2 rad52::HIS* *VII-L::TG34-HOcs-LYS2 ura3::HPH cdc13::KAN pif1-m2 pRS425-CDC13* | This study |
| DDY4625 | S288C | *MAT***a***-inc ura3-52 lys2-801 ade2-101 ochre trp1-Δ63 his3-Δ200 leu2-Δ1::GAL1:HO-LEU2 rad52::HIS* *VII-L::TG34-HOcs-LYS2 ura3::HPH cdc13::KAN pif1-m2 pRS425-CDC13* | This study |
| DDY4626 | S288C | *MAT***a***-inc ura3-52 lys2-801 ade2-101 ochre trp1-Δ63 his3-Δ200 leu2-Δ1::GAL1:HO-LEU2 rad52::HIS* *VII-L::TG42-HOcs-LYS2 ura3::HPH cdc13::KAN pif1-m2 pRS425-CDC13* | This study |
| DDY4629 | S288C | *MAT***a***-inc ura3-52 lys2-801 ade2-101 ochre trp1-Δ63 his3-Δ200 leu2-Δ1::GAL1:HO-LEU2 rad52::HIS* *VII-L::TG82-HOcs-LYS2 ura3::HPH cdc13::KAN pif1-m2 pRS425-CDC13* | This study |
| DDY4630 | S288C | *MAT***a***-inc ura3-52 lys2-801 ade2-101 ochre trp1-Δ63 his3-Δ200 leu2-Δ1::GAL1:HO-LEU2 rad52::HIS* *VII-L::TG82-HOcs-LYS2 ura3::HPH cdc13::KAN pif1-m2 pRS425-CDC13* | This study |
| DDY4631 | S288C | *MAT***a***-inc ura3-52 lys2-801 ade2-101 ochre trp1-Δ63 his3-Δ200 leu2-Δ1::GAL1:HO-LEU2 rad52::HIS* *VII-L::TG18-HOcs-LYS2 ura3::HPH cdc13::KAN pRS425-cdc13-Q256H* | This study |
| DDY4632 | S288C | *MAT***a***-inc ura3-52 lys2-801 ade2-101 ochre trp1-Δ63 his3-Δ200 leu2-Δ1::GAL1:HO-LEU2 rad52::HIS* *VII-L::TG18-HOcs-LYS2 ura3::HPH cdc13::KAN pRS425-cdc13-Q256H* | This study |
| DDY4633 | S288C | *MAT***a***-inc ura3-52 lys2-801 ade2-101 ochre trp1-Δ63 his3-Δ200 leu2-Δ1::GAL1:HO-LEU2 rad52::HIS* *VII-L::TG34-HOcs-LYS2 ura3::HPH cdc13::KAN pRS425-cdc13-Q256H* | This study |
| DDY4634 | S288C | *MAT***a***-inc ura3-52 lys2-801 ade2-101 ochre trp1-Δ63 his3-Δ200 leu2-Δ1::GAL1:HO-LEU2 rad52::HIS* *VII-L::TG34-HOcs-LYS2 ura3::HPH cdc13::KAN pRS425-cdc13-Q256H* | This study |
| DDY4635 | S288C | *MAT***a***-inc ura3-52 lys2-801 ade2-101 ochre trp1-Δ63 his3-Δ200 leu2-Δ1::GAL1:HO-LEU2 rad52::HIS* *VII-L::TG42-HOcs-LYS2 ura3::HPH cdc13::KAN pRS425-cdc13-Q256H* | This study |
| DDY4636 | S288C | *MAT***a***-inc ura3-52 lys2-801 ade2-101 ochre trp1-Δ63 his3-Δ200 leu2-Δ1::GAL1:HO-LEU2 rad52::HIS* *VII-L::TG42-HOcs-LYS2 ura3::HPH cdc13::KAN pRS425-cdc13-Q256H* | This study |
| DDY4637 | S288C | *MAT***a***-inc ura3-52 lys2-801 ade2-101 ochre trp1-Δ63 his3-Δ200 leu2-Δ1::GAL1:HO-LEU2 rad52::HIS* *VII-L::TG50-HOcs-LYS2 ura3::HPH cdc13::KAN pRS425-cdc13-Q256H* | This study |
| DDY4638 | S288C | *MAT***a***-inc ura3-52 lys2-801 ade2-101 ochre trp1-Δ63 his3-Δ200 leu2-Δ1::GAL1:HO-LEU2 rad52::HIS* *VII-L::TG50-HOcs-LYS2 ura3::HPH cdc13::KAN pRS425-cdc13-Q256H* | This study |
| DDY4639 | S288C | *MAT***a***-inc ura3-52 lys2-801 ade2-101 ochre trp1-Δ63 his3-Δ200 leu2-Δ1::GAL1:HO-LEU2 rad52::HIS* *VII-L::TG58-HOcs-LYS2 ura3::HPH cdc13::KAN pRS425-cdc13-Q256H* | This study |
| DDY4640 | S288C | *MAT***a***-inc ura3-52 lys2-801 ade2-101 ochre trp1-Δ63 his3-Δ200 leu2-Δ1::GAL1:HO-LEU2 rad52::HIS* *VII-L::TG58-HOcs-LYS2 ura3::HPH cdc13::KAN pRS425-cdc13-Q256H* | This study |
| DDY4641 | S288C | *MAT***a***-inc ura3-52 lys2-801 ade2-101 ochre trp1-Δ63 his3-Δ200 leu2-Δ1::GAL1:HO-LEU2 rad52::HIS* *VII-L::TG58-HOcs-LYS2 ura3::HPH cdc13::KAN pRS425-cdc13-Q256H* | This study |
| DDY4642 | S288C | *MAT***a***-inc ura3-52 lys2-801 ade2-101 ochre trp1-Δ63 his3-Δ200 leu2-Δ1::GAL1:HO-LEU2 rad52::HIS* *VII-L::TG66-HOcs-LYS2 ura3::HPH cdc13::KAN pRS425-cdc13-Q256H* | This study |
| DDY4643 | S288C | *MAT***a***-inc ura3-52 lys2-801 ade2-101 ochre trp1-Δ63 his3-Δ200 leu2-Δ1::GAL1:HO-LEU2 rad52::HIS* *VII-L::TG66-HOcs-LYS2 ura3::HPH cdc13::KAN pRS425-cdc13-Q256H* | This study |
| DDY4644 | S288C | *MAT***a***-inc ura3-52 lys2-801 ade2-101 ochre trp1-Δ63 his3-Δ200 leu2-Δ1::GAL1:HO-LEU2 rad52::HIS* *VII-L::TG74-HOcs-LYS2 ura3::HPH cdc13::KAN pRS425-cdc13-Q256H* | This study |
| DDY4645 | S288C | *MAT***a***-inc ura3-52 lys2-801 ade2-101 ochre trp1-Δ63 his3-Δ200 leu2-Δ1::GAL1:HO-LEU2 rad52::HIS* *VII-L::TG74-HOcs-LYS2 ura3::HPH cdc13::KAN pRS425-cdc13-Q256H* | This study |
| DDY4646 | S288C | *MAT***a***-inc ura3-52 lys2-801 ade2-101 ochre trp1-Δ63 his3-Δ200 leu2-Δ1::GAL1:HO-LEU2 rad52::HIS* *VII-L::TG82-HOcs-LYS2 ura3::HPH cdc13::KAN pRS425-cdc13-Q256H* | This study |
| DDY4647 | S288C | *MAT***a***-inc ura3-52 lys2-801 ade2-101 ochre trp1-Δ63 his3-Δ200 leu2-Δ1::GAL1:HO-LEU2 rad52::HIS* *VII-L::TG18-HOcs-LYS2 ura3::HPH cdc13::KAN pif1-m2 pRS425-cdc13-Q256H* | This study |
| DDY4648 | S288C | *MAT***a***-inc ura3-52 lys2-801 ade2-101 ochre trp1-Δ63 his3-Δ200 leu2-Δ1::GAL1:HO-LEU2 rad52::HIS* *VII-L::TG34-HOcs-LYS2 ura3::HPH cdc13::KAN pif1-m2 pRS425-cdc13-Q256H* | This study |
| DDY4649 | S288C | *MAT***a***-inc ura3-52 lys2-801 ade2-101 ochre trp1-Δ63 his3-Δ200 leu2-Δ1::GAL1:HO-LEU2 rad52::HIS* *VII-L::TG34-HOcs-LYS2 ura3::HPH cdc13::KAN pif1-m2 pRS425-cdc13-Q256H* | This study |
| DDY4650 | S288C | *MAT***a***-inc ura3-52 lys2-801 ade2-101 ochre trp1-Δ63 his3-Δ200 leu2-Δ1::GAL1:HO-LEU2 rad52::HIS* *VII-L::TG42-HOcs-LYS2 ura3::HPH cdc13::KAN pif1-m2 pRS425-cdc13-Q256H* | This study |
| DDY4651 | S288C | *MAT***a***-inc ura3-52 lys2-801 ade2-101 ochre trp1-Δ63 his3-Δ200 leu2-Δ1::GAL1:HO-LEU2 rad52::HIS* *VII-L::TG42-HOcs-LYS2 ura3::HPH cdc13::KAN pif1-m2 pRS425-cdc13-Q256H* | This study |
| DDY4652 | S288C | *MAT***a***-inc ura3-52 lys2-801 ade2-101 ochre trp1-Δ63 his3-Δ200 leu2-Δ1::GAL1:HO-LEU2 rad52::HIS* *VII-L::TG58-HOcs-LYS2 ura3::HPH cdc13::KAN pif1-m2 pRS425-cdc13-Q256H* | This study |
| DDY4653 | S288C | *MAT***a***-inc ura3-52 lys2-801 ade2-101 ochre trp1-Δ63 his3-Δ200 leu2-Δ1::GAL1:HO-LEU2 rad52::HIS* *VII-L::TG58-HOcs-LYS2 ura3::HPH cdc13::KAN pif1-m2 pRS425-cdc13-Q256H* | This study |
| DDY4654 | S288C | *MAT***a***-inc ura3-52 lys2-801 ade2-101 ochre trp1-Δ63 his3-Δ200 leu2-Δ1::GAL1:HO-LEU2 rad52::HIS* *VII-L::TG66-HOcs-LYS2 ura3::HPH cdc13::KAN pif1-m2 pRS425-cdc13-Q256H* | This study |
| DDY4655 | S288C | *MAT***a***-inc ura3-52 lys2-801 ade2-101 ochre trp1-Δ63 his3-Δ200 leu2-Δ1::GAL1:HO-LEU2 rad52::HIS* *VII-L::TG66-HOcs-LYS2 ura3::HPH cdc13::KAN pif1-m2 pRS425-cdc13-Q256H* | This study |
| DDY4656 | S288C | *MAT***a***-inc ura3-52 lys2-801 ade2-101 ochre trp1-Δ63 his3-Δ200 leu2-Δ1::GAL1:HO-LEU2 rad52::HIS* *VII-L::TG74-HOcs-LYS2 ura3::HPH cdc13::KAN pif1-m2 pRS425-cdc13-Q256H* | This study |
| DDY4657 | S288C | *MAT***a***-inc ura3-52 lys2-801 ade2-101 ochre trp1-Δ63 his3-Δ200 leu2-Δ1::GAL1:HO-LEU2 rad52::HIS* *VII-L::TG74-HOcs-LYS2 ura3::HPH cdc13::KAN pif1-m2 pRS425-cdc13-Q256H* | This study |
| DDY4658 | S288C | *MAT***a***-inc ura3-52 lys2-801 ade2-101 ochre trp1-Δ63 his3-Δ200 leu2-Δ1::GAL1:HO-LEU2 rad52::HIS* *VII-L::TG74-HOcs-LYS2 ura3::HPH cdc13::KAN pif1-m2 pRS425-cdc13-Q256H* | This study |
| DDY4659 | S288C | *MAT***a***-inc ura3-52 lys2-801 ade2-101 ochre trp1-Δ63 his3-Δ200 leu2-Δ1::GAL1:HO-LEU2 rad52::HIS* *VII-L::TG82-HOcs-LYS2 ura3::HPH cdc13::KAN pif1-m2 pRS425-cdc13-Q256H* | This study |
| DDY4660 | S288C | *MAT***a***-inc ura3-52 lys2-801 ade2-101 ochre trp1-Δ63 his3-Δ200 leu2-Δ1::GAL1:HO-LEU2 rad52::HIS* *VII-L::TG82-HOcs-LYS2 ura3::HPH cdc13::KAN pif1-m2 pRS425-cdc13-Q256H* | This study |
| DDY4678 | S288C | *MAT***a***-inc ura3-52 lys2-801 ade2-101 ochre trp1-Δ63 his3-Δ200 leu2-Δ1::GAL1:HO-LEU2 rad52::HIS* *VII-L::TG42-HOcs-LYS2 ura3::HPH cdc13::KAN pif1-m2 pRS425-cdc13-Q256H* | This study |
| DDY4679 | S288C | *MAT***a***-inc ura3-52 lys2-801 ade2-101 ochre trp1-Δ63 his3-Δ200 leu2-Δ1::GAL1:HO-LEU2 rad52::HIS* *VII-L::TG42-HOcs-LYS2 ura3::HPH cdc13::KAN pif1-m2 pRS425-cdc13-Q256H* | This study |
| DDY4680 | S288C | *MAT***a***-inc ura3-52 lys2-801 ade2-101 ochre trp1-Δ63 his3-Δ200 leu2-Δ1::GAL1:HO-LEU2 rad52::HIS* *VII-L::TG42-HOcs-LYS2 ura3::HPH cdc13::KAN pif1-m2 pRS425-cdc13-Q256H* | This study |
| DDY4693 | S288C | *MAT***a***-inc ura3-52 lys2-801 ade2-101 ochre trp1-Δ63 his3-Δ200 leu2-Δ1::GAL1:HO-LEU2 rad52::HIS* *VII-L::TG50-HOcs-LYS2 ura3::HPH cdc13::KAN pif1-m2 pRS425-CDC13* | This study |
| DDY4697 | S288C | *MAT***a***-inc ura3-52 lys2-801 ade2-101 ochre trp1-Δ63 his3-Δ200 leu2-Δ1::GAL1:HO-LEU2 rad52::HIS* *VII-L::TG50-HOcs-LYS2 ura3::HPH cdc13::KAN pif1-m2 pRS425-CDC13* | This study |
| DDY4707 | S288C | *MAT***a***-inc ura3-52 lys2-801 ade2-101 ochre trp1-Δ63 his3-Δ200 leu2-Δ1::GAL1:HO-LEU2 rad52::HIS* *VII-L::TG82-HOcs-LYS2 ura3::HPH cdc13::KAN pRS425-cdc13-Q256H* | This study |
| DDY4708 | S288C | *MAT***a***-inc ura3-52 lys2-801 ade2-101 ochre trp1-Δ63 his3-Δ200 leu2-Δ1::GAL1:HO-LEU2 rad52::HIS* *VII-L::TG82-HOcs-LYS2 ura3::HPH cdc13::KAN pRS425-cdc13-Q256H* | This study |
| DDY4712 | S288C | *MAT***a***-inc ura3-52 lys2-801 ade2-101 ochre trp1-Δ63 his3-Δ200 leu2-Δ1::GAL1:HO-LEU2 rad52::HIS* *VII-L::TG50-HOcs-LYS2 ura3::HPH cdc13::KAN pif1-m2 pRS425-cdc13-Q256H* | This study |
| DDY4713 | S288C | *MAT***a***-inc ura3-52 lys2-801 ade2-101 ochre trp1-Δ63 his3-Δ200 leu2-Δ1::GAL1:HO-LEU2 rad52::HIS* *VII-L::TG50-HOcs-LYS2 ura3::HPH cdc13::KAN pif1-m2 pRS425-cdc13-Q256H* | This study |
| DDY4714 | S288C | *MAT***a***-inc ura3-52 lys2-801 ade2-101 ochre trp1-Δ63 his3-Δ200 leu2-Δ1::GAL1:HO-LEU2 rad52::HIS* *VII-L::TG50-HOcs-LYS2 ura3::HPH cdc13::KAN pif1-m2 pRS425-cdc13-Q256H* | This study |
| DDY4715 | S288C | *MAT***a***-inc ura3-52 lys2-801 ade2-101 ochre trp1-Δ63 his3-Δ200 leu2-Δ1::GAL1:HO-LEU2 rad52::HIS* *VII-L::TG50-HOcs-LYS2 ura3::HPH cdc13::KAN pif1-m2 pRS425-cdc13-Q256H* | This study |
